# Hippocampal neurons wait their turn

**DOI:** 10.7554/eLife.02590

**Published:** 2014-03-25

**Authors:** Yuan Gao, Ian Davison

**Affiliations:** 1 **Yuan Gao** is at Boston University, Boston, United States; 2 **Ian Davison** is at Boston University, Boston, United Statesidavison@bu.edu

**Keywords:** hippocampus, CA1, trace conditioning, activity sequences, noise correlations, learning, mouse

## Abstract

Imaging large groups of neurons as mice learn to respond to an event after a time delay shows that the brain encodes the passage of time using ordered sequences of neural activity.

**Related research article** Modi MN, Dhawale AK, Bhalla US. 2014. CA1 cell activity sequences emerge after reorganization of network correlation structure during associative learning. *eLife* **3**:e01982. doi: 10.7554/eLife.01982

**Image** Noise correlation matrix showing the synchronised activity of neurons in different regions of the hippocampus

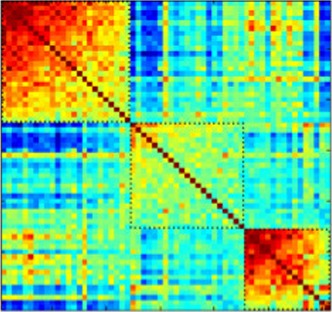

Our days are full of mental countdowns: how long until the coffee is done brewing? Until the light turns green? Until this gel has finished running? Predicting the future state of the world from the present is critical for flexible behaviour, allowing us to move beyond reflexive reactions and instead towards charting a course that minimises punishment or improves our chances of reward. We routinely link cues (e.g., coffee starts to brew) and outcomes (coffee is ready) that are separated by seconds, minutes, or longer. At the cellular level, learning involves making changes to the strength of the connections between neurons, but these changes are only triggered when two neurons are active within about 100 milliseconds of each other (Levy and Steward, 1983; Feldman, 2012). Thus, there is an apparent mismatch between the timescales for behavioural learning and neural plasticity.

How, then, does the brain link together related events that are separated in time? One possibility is that it prolongs neuron firing in some way, maintaining the neural signal from the initial cue. Several regions of the brain, most notably the frontal cortex, show persistently elevated neural activity while animals hold information in their short-term memory (Funahashi, 2006). However, simply sustaining neural activity does not carry information about how much time has passed.

Recently, recordings from a region of the brain called the hippocampus in rats have revealed ‘time cells’ that fire in repetitive sequences during the interval between an initial cue and a delayed action (MacDonald et al., 2011). By providing time information, these cells complement the well-known role of hippocampal ‘place cells’ that fire when an animal is in a specific location. It has been proposed that these neural signals for place and time help to form episodic memories that link together a series of events occurring at different locations, supplying our remembered experience (Eichenbaum, 2013).

Now, in *eLife*, Mehrab Modi, Ashesh Dhawale and Upinder Bhalla of the National Centre for Biological Sciences in India show that sequences of neural activity in the hippocampus also contribute to another form of time-based learning. Using a classic eyeblink experiment, they trained animals to blink at a certain length of time after they heard a specific tone in order to avoid a puff of air directed at their eyes (Figure 1B). Two-photon Ca^2+^ imaging during the training period revealed how neurons in the hippocampus responded as mice learnt to blink at the right time.

**Figure 1.**
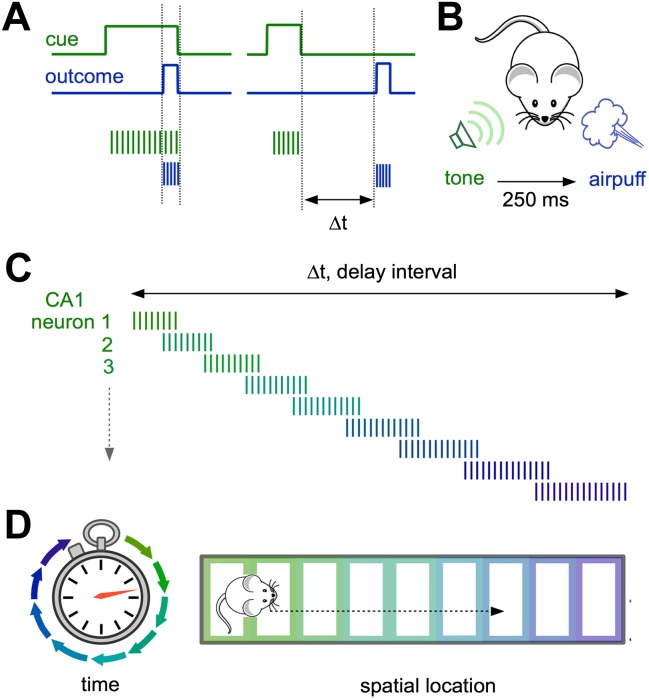
Neural representations of elapsed time in the hippocampus. (**A** and **B**) When a cue (such as a specific sound; green) and an outcome (such as a puff of air directed at the eyes; blue) overlap in time (left) and drive overlapping neural activity in different groups of neurons (vertical green and blue lines), standard plasticity processes can account for learning. However, when the cue and the outcome are separated by more than ∼100 milliseconds (right), the mechanisms for linking these events in the brain are less well understood. (**C**) Modi et al. show that the time interval between the initial cue and the predicted arrival of the puff of air is bridged by temporally ordered sequences of activated neurons in hippocampal area CA1. (**D**) Ordered sequences are a common feature of activity in CA1, and can represent the animal’s trajectory in both time (left) and space (right).

Distinct groups of cells in an area of the hippocampus known as CA1 were selectively active at each successive time point after the tone, so that their firing collectively bridged the entire delay period. CA1 therefore explicitly encodes elapsed time, with each ‘tick’ of the hippocampal clock represented by a specific set of activated neurons (Figure 1C). Importantly, reliable neural sequences emerged as the animals mastered the timing of the blink response.

While neural sequences provide an appealing explanation behind the mental stopwatch, it is unclear how they are generated in the brain. Computational work suggests that training can produce ordered sequences in appropriately connected neural networks (Goldman, 2009). The area of the hippocampus known as CA3, which provides much of the input into CA1, constitutes such a circuit. Furthermore, time cell firing in CA1 resembles the output of this model (MacDonald et al., 2011).

By looking at the activity of large numbers of neurons, Modi and colleagues provide experimental evidence that CA1 sequences are likely to result from changes in the input received from CA3. This involved assessing the noise correlations—the similarities in the random fluctuations in neural activity at rest—which arise when two neurons are either directly connected, or are both driven by the same source. Noise correlations between CA1 cells increased early in training, suggesting that their inputs from CA3 were the site of the neural changes driven by learning (Modi et al., 2014). While correlations mostly decreased again later in training, they remained high among the CA1 cells responding at the same time point, further suggesting that their final time-selectivity depends on a common source in CA3.

Similar correlation effects have been described in pairs of CA1 place cells in rats exploring a novel spatial environment (Cheng and Frank, 2008), suggesting that modifying the input from CA3 to CA1 may contribute to learning about both time and place. Interestingly, sequential activity also occurs as animals navigate through successive locations in space, driving firing in different place cells (Figure 1D).

Ordered sequences of firing are widespread in neural processing, appearing not only in other delay-based tasks (Funahashi, 2006) but also in spatial navigation (O’Keefe and Recce, 1993), complex motor actions (Hahnloser et al., 2002), and sensory perception (Shusterman et al., 2011). It will be important to understand whether these diverse contexts share common principles for generating sequences of neural firing. Since sequences are generated internally in the brain, independently of external input, identifying the region where they arise is a major goal. The findings of Modi, Dhawale and Bhalla further this effort by implicating area CA3 as this source in a task that depends solely on time.

In the future, direct measurements from CA3 itself should help to clarify its role in generating new neuron firing sequences. In spatial learning tasks, suppressing the CA3 output demonstrates that it contributes to the initial formation of new place fields, and so determines the location where CA1 place cells fire (Nakashiba et al., 2008). Similar approaches could help further define CA3’s role in learning about time intervals as well. Finally, while the delay period used here was less than a second, we often face time contingencies spanning much more extended intervals. A major remaining challenge will also be to understand how the brain tracks delays on very long timescales—such as those needed for publishing scientific papers.
